# Bioenergetic Aspects of Mitochondrial Actions of Thyroid Hormones

**DOI:** 10.3390/cells11060997

**Published:** 2022-03-15

**Authors:** Federica Cioffi, Antonia Giacco, Fernando Goglia, Elena Silvestri

**Affiliations:** Department of Science and Technology, University of Sannio, Via De Sanctis, 82100 Benevento, Italy; federica.cioffi@unisannio.it (F.C.); antonia.giacco@unisannio.it (A.G.); goglia@unisannio.it (F.G.)

**Keywords:** iodothyronines, bioenergetics, mitochondrial proteomics

## Abstract

Much is known, but there is also much more to discover, about the actions that thyroid hormones (TH) exert on metabolism. Indeed, despite the fact that thyroid hormones are recognized as one of the most important regulators of metabolic rate, much remains to be clarified on which mechanisms control/regulate these actions. Given their actions on energy metabolism and that mitochondria are the main cellular site where metabolic transformations take place, these organelles have been the subject of extensive investigations. In relatively recent times, new knowledge concerning both thyroid hormones (such as the mechanisms of action, the existence of metabolically active TH derivatives) and the mechanisms of energy transduction such as (among others) dynamics, respiratory chain organization in supercomplexes and cristes organization, have opened new pathways of investigation in the field of the control of energy metabolism and of the mechanisms of action of TH at cellular level. In this review, we highlight the knowledge and approaches about the complex relationship between TH, including some of their derivatives, and the mitochondrial respiratory chain.

## 1. Introduction

### 1.1. Respiratory Chain, Oxidative Phosphorylation

The oxidative phosphorylation system (OXPHOS, constituted by the respiratory chain and the ATP synthase complex, also named complex V), located at the mitochondrial inner membrane, is composed of two mobile transporters of electrons, coenzyme Q and cytochrome c, and five enzymes (complexes I–V), which, in mammals, are all multimeric and, except for complex II, have subunits encoded both in the mitochondrial genome (mtDNA) and the nuclear genome (nDNA). Within the chain, the respiratory complexes, organized into supercomplexes (SCs) of defined and dynamic stoichiometries [1,2], transfer electrons of reducing equivalents, extracted from energy substrates, to molecular oxygen to power the synthesis of adenosine-5′-triphosphate (ATP). Complexes I, III, and IV (proton pumps) translocate protons into the inter-membrane space giving rise to a ΔpH which, coupled to the inner membrane electric potential (Δψm), generates the proton-motive force (ΔμH^+^ or Δp), driving, in the end, ATP synthesis. 

Complex I (NAD-nicotinamide-adenine-dinucleotidedehydrogenase/ubiquinone oxidoreductase) is the first and the largest enzyme of mammalian OXPHOS. It catalyzes the oxidation of reduced nicotinamide adenine dinucleotide (NADH) by coenzyme Q and is a major site of cellular oxygen superoxide production (for recent review on the mechanisms of action of complex I, see [3,4]). Complex III (cytochrome bc1 complex; ubiquinol, cytochrome c oxidoreductase) catalyzes the oxidation of reduced coenzyme Q and the reduction of cytochrome c, which, in turn, transfers electrons to complex IV (cytochrome c-oxidase, COX) where molecular oxygen is reduced to H_2_O (for recent review on the mechanisms of action of complex III and IV, see [4,5,6]). Complex II (succinate, ubiquinone-oxidoreductase) is the smallest complex within the OXPHOS and the only one not serving to pump protons across the inner membrane. It is the second entry point of reducing equivalents into the respiratory chain: by using flavin adenine dinucleotide (FADH) as a cofactor, it couples the oxidation of succinate to the reduction of coenzyme Q (for recent review on the mechanisms of action of complex II, see [7]). The determination and analysis of the OXPHOS site-specific rates of reactive oxygen species production in mitochondria oxidizing different substrates have been reported in elegant studies by Quinlan and coworker [8]. Complex V (adenosine-5′-triphosphate synthase) utilizes the transmembrane proton-motive force ΔμH^+^, generated by respiration, to drive the synthesis of ATP from ADP and inorganic phosphate. The monomeric complex V is made up of two domains, a membrane embedded portion (so-called Fo), through which transmembrane proton translocation takes place, and a large globular soluble catalytic portion (so-called F1). As protons pass through the Fo domain, one of its portions rotates forcing F1 to act as a motor, an energy transducer, to synthesize ATP. In recent years, high-resolution electron cryo-microscopy allowed us to obtain new insights into the structural and functional interactions among the various subunits of the complex (for recent review, see [9]). Specifically, it has been demonstrated that, at each ATP-generating power stroke, the catalytic F1 head rotates with the central γ-subunit, while the flexible hinge between F1 and the peripheral stalk (subunit δ/OSCP) enables the joint rotation. Protons are conducted by subunit a through two conserved aqueous channels, separated by ∼6 Å in the hydrophobic core of Fo, where a strong local field forms generating torque to drive the rotary catalysis in F1 [6]. Electron cryo-microscopy also allowed us to highlight the role played by dimers of Complex V in shaping the inner membrane cristae [6]. Upon lipid reconstitution, purified dimers of Complex V have been shown to spontaneously form rows or ribbons that in mammalian mitochondria can measure up to 1 μm in length (e.g., in bovine mitochondria) and are able to generate membrane curvatures and, within the organelle, lamellar cristae ([2] and references within, [9] and references within).

The proton flux through the inner mitochondrial membrane is not completely coupled to ATP synthesis, giving rise to the so-called “proton leak”, which dissipates part of the energy of the H^+^ gradient as heat. Two mechanisms for proton-leak have been described so far: (1) the basal mechanism, present in most tissues and mainly related to the lipid-rich environment of the membrane [10]; (2) the inducible proton-leak, tightly regulated and occurring through specific proteins (e.g., uncoupling proteins (UCPs)) [11,12]. Proton-leak accounts for a significant part of the resting metabolic rate of an animal, represents a potential mechanism for either energy dissipation or heat production and significantly determines the efficiency of the oxidative phosphorylation, defined by the amount of inorganic phosphate (Pi) incorporated into ATP per amount of consumed oxygen, i.e., the P/O ratio [13,14]. Among the different mechanisms, a cell may engage in response to changes in energy demands to adjust ATP synthesis, the “respiratory control” is that based on feedback mechanisms that control the rate of ATP synthesis, by regulating the Δp and Δψm values. Back in 1955 [15], Chance and Williams first defined the “respiratory steady states,” in which the entire oxidative phosphorylation process could be fractionated: State 3, characterized by high respiratory chain activity and high levels of ATP synthesis; and State 4, characterized by a reduction in the respiration rate after the conversion of ADP to ATP, and an increase in Δp.

### 1.2. Thyroid Hormones, General Concepts

**Figure 1:**
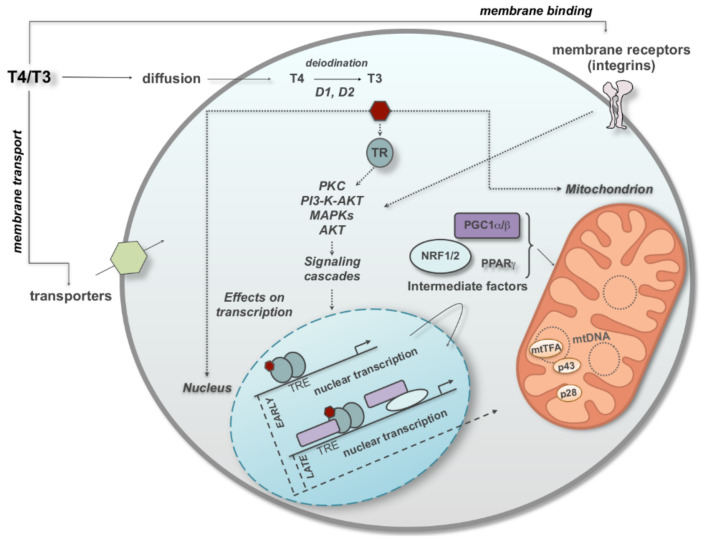
Schematic summary of the main cellular pathways through which TH regulate mitochondrial functions. TH (T4/T3) move from outside the plasma membrane into the cytoplasm (trough passive diffusion or active transport) or bind to surface receptor such as integrin αvb3. In the cytoplasm, deiodination allows the conversion of T4 into T3 by DIO1 or DIO2 action, and T3 can bind to cytosolic proteins (i.e., cytosolic TH receptors, TR). These can signal through transduction pathways involving Mitogen-Activated Protein Kinases (MAPKs), Protein C (PKC), Protein Kinase B (AKT), and phosphoinositide 3-kinase (PI3-K)-AKT, similar to those activated by integrins. All these may result in gene transcription. Direct transcriptional regulation by T3/TR requires TREs (early expression (early)). Late T3-dependent transcription (late expression (late)) requires some intermediate factors [(e.g., Nuclear Respiratory Factor 1 (NRF-1), Nuclear Respiratory Factor 2 (NRF-2), Peroxisome Proliferator-Activated Recepto (PPAR)γ, and transcriptional coactivators such as Peroxisome Proliferator-Activated Receptor Gamma Coactivator (PGC)-1α and PGC-1β) which can also enter the mitochondrion to modulate mitochondrial biogenesis, oxygen consumption, and gene expression. An important role is played by the nuclear-encoded transcription factor mitochondrial Transcription Factor A (mtTFA), a key modulator of mtDNA stabilization. Mitochondria contain two N-terminally truncated forms of the TRα1 receptor isoform, with molecular weights of 43 (p43) and 28 kDa (p28), with p43 now considered a T3-dependent transcription factor of the mitochondrial genome (adapted from [26]).

The thyroid gland produces two main types of iodothyronines: 3,5,3′,5′-tetraiodothyronine (thyroxine or T4) and 3,5,3′-triiodo-lthyronine (T3), which are considered to be the classic thyroid hormones (TH), central players in the regulation of physiological processes such as development, differentiation, growth, and metabolism in most higher organisms. It is assumed that T4 is a precursor and that T3, which is mostly (about 80%) formed by the peripheral deiodination of T4, is the active form of TH. However, some evidences suggest that other iodothyronines, such as T4 itself, and reverse T3 (rT3) as well as, other TH metabolites, such as 3-iodothyronamine (T1AM) and 3,5-diiodo-L-thyronine (T2) may possess significant biological activity [16,17,18,19]. The biological activity of T3 is largely determined by its intracellular concentration, which is dependent on its transport across the cell membrane (through transporters that belong to the monocarboxylate transporter 8, organic-anion-transporting polypeptide 1, and the L-type amino acid transporter families), and the presence of iodothyronine deiodinases (DIOs) and other enzymes such as decarboxylase and deaminase [16,20,21,22,23]. DIO1 and DIO2, the so-called activating DIOs, convert intracellular T4 to T3, while DIO3, the so-called inactivating DIO, converts T3 to rT3. The mechanisms of the hormonal action at cellular level can be divided in genomic and nongenomic. Genomic mechanisms consist of modulating the transcription of specific genes which is mediated via nuclear uptake of the T3, the formation of complexes between T3 and nuclear thyroid hormone receptor (TR) proteins, and the subsequent occupancy of regulatory complexes (composed of TR and other nucleoproteins) at thyroid hormone response elements (TRE) on hormone-responsive genes. TRs are comprised of two major isoforms, TRα1 and TRβ1, encoded by two separate genes, THRA and THRB, respectively, with TRα1 being predominantly expressed in bone, cardiac, skeletal muscle and the gastrointestinal tract, whereas TRβ1 is expressed in the liver and kidney [24]. Nongenomic mechanisms, on the other hand, are independent of nuclear uptake of T3 and intranuclear formation of T3–TR complexes with other nucleoproteins and are initiated in the plasma membrane, in cytoplasm, or in mitochondria. Receptorial proteins involved in nongenomic actions may or may not be homologues to TRs [25]. The effects exerted by T3 on mitochondria may involve both of these mechanisms and a general schematic representation is shown in Figure 1. 

## 2. Thyroid Hormones and the OXPHOS

Historically, to study the mechanisms underlying the alterations produced in mitochondrial functions by TH and the extent to which components of the respiratory chain limit respiration, in 1995, Harper and Brand introduced the metabolic-control analysis [27], according to which the OXPHOS can be divided into “blocks” of reactions: (i) those involved in the oxidation of substrates and generating ΔμH^+^ (i.e., catalyzed by substrate translocase, substrate dehydrogenase, and components of the electron transport chain); (ii) those dissipating ΔμH^+^ for the synthesis and export of ATP (i.e., involving ATP synthase, the phosphate transporter, and the adenosine nucleotide translocase (ANT)); and (iii) those dissipating ΔμH^+^ without ATP synthesis, before mentioned as “proton leak” reactions. Each block exerts a different type of control over respiration, with State 4 principally controlled by the leakage of protons across the mitochondrial inner membrane, and State 3, on the other hand, mainly controlled by changes in the activity of the phosphorylation system [28]. Following this approach, Hafner et al. performed the pioneering studies examining the kinetic responses of ΔμH^+^ producers and ΔμH^+^ consumers in rat liver mitochondria during the changes that occur from the euthyroid to hypothyroid and from the euthyroid to hyperthyroid states [29,30]. For the first time, results suggested that different mechanisms might operate in the hypothyroid and hyperthyroid states. Specifically, in the transition from euthyroidism to hypothyroidism, the involvement of ANT was suggested. Accordingly, it was reported that both ANT activity and the cytosolic and mitochondrial ADP/ATP ratios are affected by the thyroid state [31,32] and that T3 injection into hypothyroid rats induces a twofold increase in liver mitochondrial ANT content and a threefold increase in its activity [33]. State 3 respiration could also be increased by T3 due to a greater amount of mitochondrial calcium uptake, which, in turn, could increase mitochondrial dehydrogenase activity [34]. In the transition from euthyroidism to hyperthyroidism, increased electron flow through the respiratory chain can result from either increased amounts of components of the respiratory chain or an increased reduction state. Indeed, T3 can directly or indirectly activate the transcription of nuclear and mitochondrial respiratory genes [35,36,37] and increase the amount of reduced cytochromes [38]. THs, key modulators of metabolic efficiency [39,40] (and references within), can also affect State 4, mainly activating proton leak, which is reported to be depressed in hypothyroid mitochondria and stimulated in hyperthyroid mitochondria [41]. 

### Thyroid Hormones and the Mitochondrial Efficiency

According to the so-called “uncoupling hypothesis”, the TH-induced stimulation of energy expenditure, the universally recognized calorigenic effect of TH [42], is achieved, at the mitochondrial level, through uncoupling the electron transport chain from ATP synthesis, thus reducing the P/O ratio [43,44]. Historically, in early studies, the above effects were detected in vitro and only at pharmacological doses of TH [43,44], thus being considered insignificant and, somehow, chemical artifacts. Later, after fifty and more years of research, using different animal and in vitro models have brought this vision back to the fore and provided mechanistic, genetic, biochemical and molecular information.

To date, to explain the molecular basis of TH-induced metabolic inefficiency, two mitochondrial mechanisms have been proposed: effects on proton leak and effects on redox proton pumps, the so-called “redox slipping”. The latter mechanism refers to the failure of the proton pumps occurring when they transfer electrons with a reduced extrusion of protons out across the membrane, and a higher respiratory rate is necessary for a certain level of ATP synthesis to be maintained. In vitro, it has been demonstrated that proton-leak and redox-slipping differentially affect basal respiration and the individual contributions significantly depend on the temperature at which mitochondria are incubated. For further review, see [26]. 

As far as it concerns the proton leak, the major process that controls mitochondrial oxygen consumption when cellular ATP requirements are low, it has been measured in mitochondria from various tissues. Physiologically, both in liver and skeletal muscle, it accounts for a high proportion of the cellular metabolic rate and in liver, it may increase in a TH-dependent manner. Although known for many years, the precise mechanism whereby proton leak occurs is still under investigation. The involvement of specific inner membrane proteins was hypothesized after obtained evidences according to which variation in the fatty acid composition of mitochondrial-membrane phospholipids reconstituted in liposomes did not account for the difference in the magnitude of proton conductance [45]. 

The paradigm of the mechanisms of inducible proton-leak, which, today, we know to require activation of mitochondrial anion carrier protein function, resides in brown and beige mammalian adipose tissues, where H^+^ leak across the inner mitochondrial membrane is facilitated by UCP1, which co-transports H^+^ with short chain fatty acid (FA) anions into the mitochondrial matrix [46,47], and is inhibited by guanosine diphosphate [12]. In other tissues, not expressing UCP1, H^+^ leak can be carboxyatractyloside-sensitive, implicating the ANT family of proteins [48] or involving the permeability transition pore (PTP) (for review, see [38]). By both genomic and non-genomic actions, TH plays an important role in modulating both UCP- and non-UCP-mediated proton leaks. 

In brown adipose tissue (BAT), a time- and tissue-specific modulation of TH action on mitochondrial UCP1-dependent uncoupling, is assured by a signal pathway involving a fine regulation of the intracellular concentration of T3, by type 2 deiodinase (DIO2), driven by the β-adrenergic cyclic AMP (cAMP) signaling cascade promoting the conversion of the prohormone T4 into T3, and, ultimately, positively regulating UCP1 transcription. This pathway basically requires the interplay of both the α and β isoforms of TR [49] and a modulation of substrate availability for mitochondrial oxidation through an activation of lipolysis of BAT triglycerides. In recent years, it has been demonstrated that TH can also increase UCP1 expression in subpopulations of white adipocytes interspersed within the subcutaneous white adipose tissue (WAT), e.g., during prolonged cold exposure, inducing the so-called browning of WAT to increase the thermogenic capacity and maintain body temperature [50,51]. 

Based on sequence identity, other structurally-UCP1-related proteins with putative uncoupling properties have been described in tissues other than adipose ones. UCP2 and UCP3 are the most well-studied and their transcription is induced by TH [52,53,54,55,56,57]. UCP2 is ubiquitously expressed and has been suggested to catalyze proton transport in the direction of the proton gradient when activated by reactive oxygen species (ROS) or lipid peroxidation products [58]. UCP3, predominantly expressed in skeletal muscle, and to a lesser extent in heart, BAT, and WAT, has been associated with TH-induced modulation of resting energy expenditure [55,59] and fatty acid peroxide-induced mitochondrial uncoupling [60]. Two research groups, Costford et al. [61] and Lombardi et al. [60], using mitochondria isolated from UCP3-knockout mice and wild-type littermates, showed that UCP3 is involved both in mediating the translocation of lipid hydroperoxide across the inner mitochondrial membrane and in lipid hydroperoxide-dependent mitochondrial uncoupling. These studies further supported the hypothesis of an involvement of the homologs of UCP1 in the modulation of ROS production and oxidative stress [62,63]. Additional actions of uncoupling proteins have been extensively reviewed elsewhere [26,39].

In the liver, the uncoupling of the oxidative phosphorylation and the FA sensitivity of the proton leak have been attributed to ANT proteins. Early studies demonstrated that T3 could rapidly increase the activity of ANT in intact rat liver and in isolated mitochondria [32]. Moreover, TH, when administered to hypothyroid rats, increase the ANT content of liver mitochondria by twofold [31,33]. Thus, the TH-induced uncoupling observed in the liver can be explained by a hormone-induced increase of ANT activity/concentrations, which, in turn, involves an elevation of the FA-mediated mitochondrial proton conductance. 

As stated above, mitochondrial proton leak may further be affected by the PTP [64,65,66,67,68,69]. Starting with the studies from the 1950s and 1960s showing the ability of TH to induce mitochondrial swelling by activation of water transport to mitochondria [70,71,72], in the last 30 years, a great research effort has been made to uncover the molecular mechanism through which TH can regulate the PTP gating [73,74,75,76,77]; however, this still remains not completely elucidated (for review, see 39). One reason is the fact that PTP has so far been defined only through its function (i.e., as Ca-activated cyclosporine-inhibited mitochondrial swelling), while its structural arrangement has yet to be precisely described, with the number of its protein subunits continuously increasing [78,79,80] while some of them are being dismissed [81,82]. Additionally, the main method used for evaluating the PTP function is more than 60 years old with only a few recent attempts of amelioration [77,83]. Nevertheless, the data obtained so far indicate the PTP as a potential mitochondrial target of T3-induced uncoupling. In one of the proposed model, the gating of PTP by TH may present itself as either a low-conductance or a high-conductance gating (based on the cell energy requirements), with the underlying transduction pathways converging on the mitochondrial activity of the Bcl2-family proteins: TH would induce the efflux of calcium ions from the endoplasmic reticulum, followed by dephosphorylation of mitochondrial Bcl2(S70) by the calcium-activated protein phosphatase 2B(PP2B)/calcineurin and increase in mitochondrial Bax, for extensive review, see [39].

All this is very important when considering the recent evidences of the involvement of the mitochondrial PTP in the pathogenesis of diseases such as cardiomyopathies, neuropathies, and liver diseases [84,85,86], as well as in the process of ageing [87] and the need to design new targets and treatments to modulate mitochondrial uncoupling and metabolic efficiency. A summary of the effects elicited by TH on the mitochondrial respiratory chain is reported in Table 1.

## 3. Nongenomic Regulation of Mitochondrial Respiratory Chain by TH with a Look at the Effects of T2

Several in vivo and in vitro studies have reported rapid effects of TH on mitochondrial oxygen consumption and OXPHOS, measurable within 2–30 min after hormone administration [91,92,93,94,95]. Historically, the detection of specific T3-binding sites within mitochondria [96,97,98] suggested the existence of direct mitochondrial actions of T3 (i.e., nongenomic actions of T3). In addition, it is now recognized that other iodothyronines and TH metabolites with low or no binding affinity for nuclear TRs, can exert relevant mitochondrial effects underlying significant metabolic adjustments, for reviews, see [16,18,19,99,100,101]. Among these, the naturally occurring iodothyronine T2 and the endogenous thyronamine, T1AM, are attracting increasing interest.

**Figure 2:**
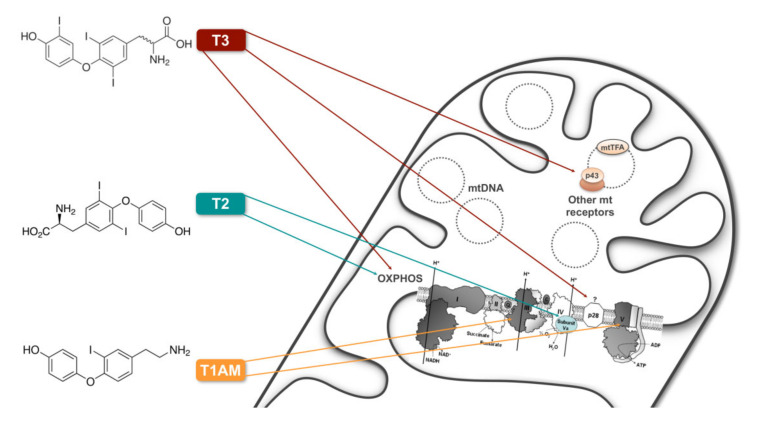
Nongenomic actions of T3 and its endogenous metabolites, T2 and T1AM, on mitochondrial respiratory chain. Cross-section of a mitochondrion (mt), with a schematic view of OXPHOS. I = Complex I; II = Complex II; III = Complex III; IV = Complex IV; V = Complex V. Arrows indicate the so far identified molecular targets of the three molecules. T3, through p43, affects mitochondrial transcription and protein synthesis and the overall activity of the OXPHOS. T2, like T3, is able to modulate the OXPHOS and to directly stimulate Complex IV (COX) activity, likely specifically binding the Va subunit. T1AM was demonstrated to modulate Complex V kinetic properties and to partially block Complex III. Mitochondrial actions of T3 mediated by p28 largely remain unknown (?).

As far as it concerns the mitochondrial T3-specific binding sites, it has been demonstrated that the full-length mRNA for the nuclear receptor isoform TRα1, through alternative start sites encodes two ubiquitously expressed additional proteins both imported into mitochondria named, based on their molecular masses, p43 and p28 (for recent review, see 93 and references within). Both displaying T3 binding affinity (being the calculated Ka values 3.3 × 10^10^ M^−1^, 2 × 10^9^ M^−1^, and 3 × 10^9^ M^−1^ for p28, p43 and TRα1, respectively), while p28 associates with the inner mitochondrial membrane, p43 localizes to the matrix [97,98,99,100]. Here, p43, binding to T3 responsive elements in the organelle genome, promotes, in the presence of T3, mitochondrial transcription and the synthesis of mitochondrial encoded proteins (Figure 2). If p28 activity remains largely unknown [102], p43 has been reported to be central to stimulate mitochondrial respiratory chain and modulate the mitochondrial/nuclear cross talk to finally affect cell proliferation and differentiation, oncogenesis, apoptosis and, in skeletal muscle, metabolic and contractile phenotype, and glycaemia regulation [103,104,105,106,107,108,109,110,111].

Some decades ago, it was shown that T2, at very low concentrations (pM range), was able to stimulate oxygen consumption in perfused livers isolated from hypothyroid rats [112], the effect being more rapid than that of T3, and DIO1 activity- as well as cycloheximide-independent, suggesting that the nucleus and deiodination did not contribute, unlike what was evident for T3 [112]. These results together with those showing a direct interaction of T2 with mitochondria [96], led to the idea that mitochondria and bioenergetic mechanisms may be the major targets of T2 within the pathways underlying its ability to stimulate cellular respiration. Later studies demonstrated that T2 (i) stimulates mitochondrial respiratory activities [113,114,115,116,117], (ii) rapidly and directly stimulates bovine heart-isolated COX [118], (iii) specifically binds the Va subunit of the COX complex (maximal stimulation of ATP-inhibited activity by 130% being obtained at 10 µM cytochrome c with 10^−6^ M T2) [119] (Figure 2), and (iv) by binding to COX, abolishes the allosteric ATP inhibition of the complex, decreasing its respiratory control ratio and leading to heat production [120]. Of note, these last-mentioned studies preceded subsequent ones that revealed the existence, in COX, of a steroid binding site conserved from Rhodobacter sphaeroides to mammals with high affinity for the bile salt deoxycholate [121], paving the way for the conceptualization of a COX regulatory site for steroids. Indeed, more recently, estradiol, testosterone, other steroids and THs have been shown to bind to and inhibit bovine heart purified COX [122,123]. Specifically, as far as it concerns THs, the existence of two separate sites of COX interaction, differing in location, affinity and specificity to hormone binding, has been reported [123], one of the two presumably being the point of T2 binding on the Va subunit described in [119].

By a top-down elasticity analysis, Lombardi and coworker showed that T2, 1 h after its injection into euthyroid rats (at the dose of 150 µg/100 g body weight), stimulates the hepatic activity of both cytochrome c-oxidizing and -reducing components of the respiratory chain [124]. A subsequent study by Cavallo and colleagues [125] reported that in liver of hypothyroid rats, within 1 h after its injection, at the same dose used by Lombardi and coworker [124], T2 affects complex V synthetic and hydrolytic activity, with no change in complex V b-subunit mRNA accumulation or a-b subunit protein amounts, likely acting at the level of cardiolipin (CL) [126]. On the other hand, when chronically injected into hypothyroid rats, T2 (at the dose of 15 or 25 μg/100 g body weight) can upregulate protein levels of complex V subunits [89,90], with activation of the transcription of a-subunit of GA-binding protein/nuclear respiratory factor-2, which has been suggested to be the mediator of the T2-transcriptional activity [89]. After chronic administration into hypothyroid rats, T2 (at the dose of 25 μg/100 g body weight) has also, more recently, been shown to increase the abundance of glycerol-3 phosphate dehydrogenase and activities of complex I and complex II measured in gel by Blue-Native polyacrylamide gel electrophoresis (BN-PAGE) [90].

When administrated to rats fed a high fat diet (HFD), T2 has been reported to prevent body weight gain, liver steatosis, hypercholesterolemia, hypertriglyceridemia and to rapidly (within 6 h) stimulate hepatic fatty acid oxidation via activation of sirtuin 1 (SIRT1), leading to deacetylation of the SIRT1 targets PGC-1α and sterol regulatory-binding proteon-1c with a downstream upregulation of genes involved in mitochondrial biogenesis and a concomitant downregulation of lipogenic genes [127]. In the same animal model, by BN-PAGE analysis, T2 has been shown to partially restore respiratory complex I and II levels and increase the activity of all respiratory complexes (except that of complex V) [128,129]. In rat skeletal muscle, T2 has resulted to stimulate mitochondrial uncoupling [126,130]. Interestingly, metabolic and lipid-lowering effects of T2 have also been shown in mice [131,132,133]. 

In in vitro models of non-alcoholic fatty liver disease, i.e., rat primary hepatocytes and hepatoma cell lines (FAO), these last defective for functional TRs, both rendered “fatty” by exposure to an oleate/palmitate mixture, T2 (concentrations ranging 10^−7^ to 10^−5^ M) was able to reduce lipid excess and either stimulate carnitine palmitoyl-transferase expression and COX activity [134], or induce mitochondrial uncoupling [135]. In addition, in pituitary GH3 cells, Del Viscovo et al. [88] demonstrated that T2 (10 × 10^−9^ M) exerts short-term effects on intracellular calcium [Ca^2+^]i and nitric oxide via plasma membrane and mitochondrial pathways. Moreover, by interacting with OXPHOS, T2 activates the mitochondrial Na^+^/Ca^2+^-exchanger [88].

Besides the newly discovered TH derivates, T1AM, originally discovered as an activator of trace amine-associated receptor 1 (TAAR1, a membrane spanning G-protein coupled receptor), is now considered an endogenous hormone-like molecule involved in a number of different biological systems, being able to induce dramatic decreases in core temperature, heart rate, and cardiac output in mice [136], all opposite actions vs. those elicited by T3, suggesting it might also counteract the classical actions mediated by TH, likely operating in a complex system of either overlapping or antagonistic effects in the regulation of metabolism (for recent review, see [137]). The first identified intracellular effector of T1AM action in vitro was the mitochondrial Complex V. At low, endogenous, concentrations (ranging in rat tissues around 10–90 nM), T1AM was demonstrated to positively affect mitochondrial energy production, by modulating Complex V kinetic properties [138] (Figure 2). When applied to rat liver mitochondria, T1AM was observed to reduce mitochondrial O_2_ consumption and increase H_2_O_2_ release), the effect likely due to a partial block of Complex III at a site located near that of action of antimycin A [139] (Figure 2). More recently, in pancreatic beta MIN6 cells, at low submicromolar concentrations, T1AM resulted in a significant reduction of insulin secretion through decreased mitochondrial ATP production [140].

Importantly, while T1AM does not interact with both canonical and noncanonical TH cellular receptors, a lively debate still exists on the possibility that T2 might exert some of its effects in a TR-dependent manner. Indeed, the binding affinity of T2 for TRs, previously reported to be 500–1000-fold lower than that of T3 in the rat [141], has been recently revisited in particular for human and teleostean TRβ1s, with a specific teleosts (long) TRβ1 isoform showing equal affinity for T2 and T3 [142].

## 4. Iodothyronines and Respiratory Supercomplexes

The structural organization of OXPHOS in the inner mitochondrial membrane is attracting more and more scientific attention as it is evident that its dynamics may be central for the regulation of bioenergetic functions as well as of mitochondrial ultrastructural and macro reorganization under patho-physiological stress or variations in the availability of nutrients or oxygen and in response to hormonal stimulation or depletion. To date, it is intended in terms of two paradigms: (1) the so-called “fluid” model, according to which all redox components are unconstrained diffusible particles [143]; (2) the so-called “solid” model, in which the respiratory complexes establish interactions to each other to form higher-order supramolecular structures called SCs (or respirasomes). The “core” of these structures contains Complex I, Complex III and Complex IV and has been shown to be conserved in all higher eukaryotes. SCs may also assemble into megacomplex where more than one unit of Complex I, Complex III, and Complex IV are together [144]. Based on BN-PAGE, Complex II does not assemble in SCs [145]. Since the “solid” model was proposed, the optimal performance of the electron transfer chain is now conceived, integrating the two models, as the result of the balance between individual mitochondrial respiratory complexes and SCs, with CoQ and cytochrome c both trapped into SCs and freely diffusible, giving raise to the “plasticity” model [146], which, however, has not yet been proven in vivo. Confirmation of SC assembly has been provided by X-ray crystallography and, more recently, by electron cryo-microscopy ([147,148,149], for recent review, see [150]) which allowed us to determine high-resolution 3D structures of a wide range of SCs from different organisms [1]. Recently, it has been shown that the interaction of respiratory complexes within mammalian mitochondrial SCs leads to changes in the individual complex structure suggesting functionally relevant structural crosstalk between the complexes [151]. In the same study a ’quinone tunnel’ has been observed linking a unit of Complex I and two units of Complex III (CI-CIII2). Specifically, the quinone tunnel has been shown to connect CI with only one monomer of CIII2, suggesting an asymmetric structural and functional arrangement of the CI–CIII2 supercomplex, which, although its in vivo relevance has yet to be elucidated, further supports a functional role for SCs in mitochondria [152]. For a recent review on mitochondrial SCs and their pathological implications, see [2].

As for today, it remains unclear which factors (included hormones) determine the structure and stoichiometry of the respiratory supercomplexes. Membrane lipid content, composition, and peroxidation seem to be central players. A special role of CL in the SCs stabilization and of phosphatidylethanolamine in the SCs destabilization has been suggested [153,154,155,156,157,158]. Membrane potential and protein phosphorylation also play roles in such processes. Data in the literature additionally suggest the involvement in SCs assembly of interacting proteins such as ANT [159,160] and UCP3 [161].

Functionally, the assembly of SCs has been proposed to have several biological significances, among which are: reduction of ROS generation [162,163]; optimization of the catalytic activity of the individual complexes [164]; boost of the efficiency of electron transfer through substrate channeling [145,149,165]; stabilization and assembly of Complex I [166,167]; prevention of aggregation of protein subunits of SCs themselves [168]. Some of these functions are still under debate (for recent review, see [169]). 

Taking into account the pleiotropic effects which TH may evoke in the mitochondrial compartment, under both physiological and pathological states, affecting quali- and quantitatively, respiratory complexes, energy efficiency, and membrane lipid composition [170,171], it should not be surprising that a close interrelation between THs and SCs could exist (Table 1). Unfortunately, to date, very few studies have been devoted to this specific matter. During the last decade, some new insights into both the mitochondrial response mechanisms to iodothyronines and the resulting proteome alterations have been obtained by applying one-dimensional BN-PAGE to examine the abundance of mitochondrial OXPHOS complexes as well as their activity and supramolecular organization [90,128,172,173,174]. By this approach, the influence of the administration of either T2 or T3 to chemically induced hypothyroid rats on the organization of OXPHOS into SCs, in liver mitochondria, was studied [90]. The results indicated differential effects elicited by the two tested iodothyronines: T2, influences the kinetic properties of specific mitochondrial respiratory pathways, and promotes a rapid response of the organelle; T3, enhances the abundance of respiratory chain components and favors the organization of respiratory chain complexes in SCs, inducing a slower and prolonged response. Specifically, T2 enhanced both coupled and uncoupled respiration in succinate-energized liver mitochondria, but the effect on uncoupled respiration did not involve proton leak, as in the case of T3. Moreover, different from T3, T2 was not able to influence the abundance of respiratory complexes but it enhanced the activity of complex II-linked mitochondrial respiratory pathways and the “in gel activity” of complex II. T2 also stimulated G3PDH activity, suggesting the existence of a selective effect of T2 on mitochondrial pathways producing FADH2, that could represent an iodothyroinine-specific mitochondrial thermogenic mechanism, considering that the amount of ATP generated by FADH oxidation is lower than that generated by NADH oxidation at complex I. T3, on the other hand, while sharing with T2 the ability to activate complex II- and G3PDH-linked respiratory pathways and increase complex V and G3PDH levels (being its effects more pronounced that those of T2), significantly activated proton-leak and increased ANT mitochondrial levels, both clear cut signs of its potent thermogenic action. Finally, only T3 favored the assembly of individual respiratory complexes in SCs, this likely being a mechanism to enhance the tissue’s oxidative capacity, sustain the increased metabolic rate, and limit or balance ROS generation under hyperthyroid conditions [90].

## 5. Iodothyronines, Mitochondrial Dynamics and Mitophagy

The link between form and function finds its full realization in mitochondrial architecture. Over the past decades it has universally been accepted the ideas that mitochondria are not static but very “dynamic” organelles, since they can change themselves at macro-and ultrastructural levels. They are able to respond cellular cues and energy demands by remodeling their morphology and shape, regulating directly their bioenergetic functions, allowing the cell to respond to its ever-changing physiological conditions [175,176,177,178]. Mitochondria can exist in networked phase or in small units [179,180], undergoing constant turnover (mitophagy-biosynthesis) and continuously fusing (fusion) and dividing (fission) [181,182,183,184,185].

Ultrastructurally, cristae represent the high specialized areas where OXPHOS occurs and assembles in respiratory complexes and SCs [186,187]. Cristae are membrane invaginations protruding into the mitochondrial matrix perpendicularly to the inner boundary membrane, i.e., the unfolded portion of the inner mitochondrial membrane. The cristae interior is called intracristal space and communicates with the outer intermembrane space through the cristae junction, which divides the cristae membrane from the inner boundary membrane (for review see, [188]). A major component of the cristae junction is the mitochondrial contact site and cristae organizing system (MICOS), composed of two distinct MICOS subcomplexes marked by the core components Mic60 and Mic10 (for review, see [189]).

Cristae shape and composition change under physiological stimuli, impacting profoundly on mitochondrial performance and survival [190]. From the first observation of the cristae dynamism [191], over the years, many more studies have confirmed inner mitochondrial membrane plasticity [192]. As recently shown, individual cristae differ not only in their composition [193] but also in their functionality [194]. Cristae fold increases under energy pressure, to augment oxidative phosphorylation areas [195], under the orchestration of several classical (i.e., TH) and newly discovered “remodeling” master regulators, namely: (i) Optic atrophy protein (OPA1), [196], (ii) MICOS [197], (iii) Complex V [198,199]. 

At the macroscopic levels, under high energy demands, the shift towards fusion favors the generation of interconnected mitochondria, for maintaining normal mitochondria health, by mixing healthy and damaged organelle as complementation [200], and for increasing respiratory capacity. Indeed, mitochondria “hyperfusion” is accompanied by increased mitochondrial ATP production, to optimize mitochondrial function [201]. In contrast, a shift towards fission produces small mitochondria, functionally distinct small spheres or short rods. 

Recent evidences support the idea of a tight interplay between mitochondrial dynamics and autophagy as collaborating actors in the same quality control system. Mitochondrial division or loss of fusion membrane protein (i.e., mitofusin 2 (Mfn2) or OPA1), can drive the removal of damaged organelles from the mitochondrial network to allow a specific form of autophagy, referred as mitophagy [202]. Although mitophagy shares many of characteristic aspects with autophagy [203], the selectivity of mitochondrial cargo by the autophagosome is driven by a specific protein set and stimuli [202]. 

The modulation of cristae organization, mitochondrial dynamics and mitophagy al together deeply impact mitochondrial activity. These processes are among those tightly regulated by thyroidal state. Early studies by electron-microscopy showed that T4 treatment increases cristae in the number and length, by inducing a hypermetabolic state in skeletal muscle of both normal and thyroidectomized rats [204]. Recently, electron microscope analyses indicate that increased respiratory enzyme activity produced by TH in the liver is, in large part, the result of enhanced development of the cristae folding membrane [205]. Conversely, the depression of respiration found in thyroidectomized or hypothyroid animals is correlated to a decreased cristae area [206]. In the developing brain, T3 deprivation determines highly fractured and degenerated mitochondrial cristae with low membrane potential, due to the decreased membrane surface area rather than of enzymes of the respiratory chain, with compromised OXPHOS [207].

Until now, there are no mechanistic studies that correlate directly TH administration with fusion/fission processes. Very recently, it has been shown how T2 modulates mitochondrial dynamics in skeletal muscle of HFD-fed rats. In particular, T2 treatment inhibits diet-dependent mitochondrial shift toward fission, protecting muscle cells against mitochondrial dysfunction [208]. 

In regard to quality control system, the ability of TH to activate several forms of autophagy (from mitophagy to lipophagy) has been finely investigated [209,210]. To counteract ROS-inflicted cell damage and death, T3 induces mitophagy via Beclin1, PTEN Induced Kinase (Pink)/Parkin (Park), and Microtubule-associated protein 1A/1B-light chain 3 (LC3) pathways [211,212,213,214]. However, the coupling of mitophagy and OXPHOS [213,214] occurring during TH-regulated metabolism, can be enrolled not only to repair mitochondrial damage, but also to prevent it and maintain high energy demand. In liver of mice, T3 induces mitophagy via the ROS-AMP activated protein kinase (AMPK)-unc-51-like autophagy activating kinase 1 (ULK1) pathway [210] that primarily reduces ROS damage accumulation, promoting mitochondria renewal. Sinha and co-workers demonstrated, in vivo/in vitro, that mitophagy is necessary also for sustaining efficient oxidative phosphorylation induced by T3, during times of high-energy demand [210]. Moreover, both T3 and T2 have been shown to be able to induce in liver autophagy and increase the mobilization of lipids (lipophagy) from lipids droplets towards beta oxidation to provide substrates for OXPHOS, maintaining a hypermetabolic state [210,213]. In soleus of hyperthyroid mice, T3-mediated mitochondrial activity and biogenesis are also linked to autophagy activation via ROS-AMPK-ULK1 in a sort of positive loop that improves mitochondrial function [214]. A similar effect has been recently found also in brown adipose tissues and brown primary preadipocytes from hyperthyroid mice, where T3 has been reported to act on mitochondrial respiration, OXPHOS as well as autophagic flux, mitophagy, and mitochondrial biogenesis, the trigger of mitophagy likely being the mTOR inhibition [215]. 

All these data corroborate the universal recognition of the intimate connection between TH and mitochondrial dynamism to improve bioenergetic performance. However, more efforts have to be made to well-characterize the direct impact of THs on such specific mechanisms, since the literature is still poor. 

## 6. Thyromimetics and Mitochondria

Several thyromimetics, besides the natural occurring TH derivatives, have been designed to specifically avoid binding to TRα while maintaining their affinity to TRβ isoforms [16] and references therein, thus uncoupling beneficial actions on liver and central nervous system from deleterious effects on the heart, muscle and bone. Among these, GC-1 (also known as sobetirome), a halogen-free thyroid hormone agonist, was the first TRβ selective thyromimetic to be developed. It has been reported to increase mitochondrial O_2_ consumption and H_2_O_2_ production but to a lesser extent compared to T3 putatively due to the higher increases induced by T3 in NRF-1 and 2 levels [216]. Even though showing encouraging actions against hypercholesterolemia, non-alcoholic steatohepatitis and in the stimulation of hepatocytes proliferation, it was stopped after phase 1 clinical trials [217]. Another agent being studied is VK2809 (formerly known as MB07811) which exhibits increased TR activation in liver relative to other tissues ([16] and references therein) and is now considered one of the most promising lipid-lowering agent, currently under phase 2–3 clinical trials. VK2809 has been reported to be able to augment metabolic rate in the liver and, specifically, increase the rate of mitochondrial FA oxidation, of mitochondrial respiration as well as the activity of hepatic mitochondrial glycerol-3-phosphate dehydrogenase [218]. Furthemore, VK2809 treatment has been reported to decreased hepatic triglyceride levels in glycogen storage disease type Ia mice, through a simultaneous restoration of autophagy, mitochondrial biogenesis, and FA oxidation [219]. Mechanistically, most of the effects caused by these thyromimetics depend on their binding to TRβ receptors, however, at least for CG1, non-genomic effects have also been reported, the latter requiring its binding to the TH membrane receptor integrin alfavbeta3 [220]. 

In recent times, also functional rather than structural TH analogs, with lower affinities for TRs, were designed and synthesized. Among these, substituted pyrazoles such as TRC150094 (TRC), which has attracted particular attention emerging as a novel functional analog of iodothyronines linking fat consumption with the pathogenesis of hepatic steatosis [221]. In particular, in a preclinical animal study, TRC has been demonstrated to stimulate mitochondrial FA uptake and oxidation as well as OXPHOS activity [221]. For these reasons it is now considered a novel mitochondrial modulator, although its mechanism of action as well as its cellular receptors have not yet been identified. Recently its safety, tolerability, and pharmacokinetics after oral administration to overweight/obese adult and elderly subjects have been studied in a randomized, Phase-I clinical trial [222]. Moreover, a methyl-substituted dicarboxylic acid thyromimetic and its analogs have been reported to directly target mitochondrial PTP gating, thus showing a thyromimetic/calorigenic activity in vivo [76].

## 7. Conclusions

As stated in a previous paper: “It would be convenient, not to say desirable, to explain the various actions of TH at the cellular level by a unitary molecular model. However, while this is an attractive idea, the bulk of available evidences indicates that the situation is far more complicated” [223] (p. 119) and still a lot of work has to be performed to fully understand the complex mode of action of THs, in terms of involved mechanisms and cellular targets. It seems universally accepted that iodothyronines may affect mitochondrial activity by two pathways, the first way is ‘nuclear-mediated’ (genomic) and involves a direct interaction of TH with nuclear receptors, while the second way requires a direct interaction of the hormones with the cellular organelles or the molecular components. The first pathway, however, does not exclude the possibility that, although the nuclear pathway is not directly involved, the latter can be recruited through signals starting from the primary site of interaction of the TH. As previously described, almost all components of the respiratory chain are directly or indirectly affected by iodothyronines (see, Table 1). In some cases, the actions result in an activation of specific biochemical pathways while in others the effects would result into an increase in mRNA or protein levels of specific components of respiratory chain. Another obstacle for a clear and unambiguous explanation of the mechanisms activated by iodothyronines is that they can exert both short-term (in hours) and long-term (days) effects. So, given this, in animal studies the duration of the hormonal treatments is crucial to observe some effects. In addition, as often isolation of mitochondria is required, it is crucial to compare the methods of separation of the organelles. In fact, in various studies, mitochondria are often isolated at different gravitational forces (ranging from 3000 g to 12,000 g). In these cases, each different preparation results in mitochodria endowed of biochemical and functional differences. This was evident in a study performed on rat liver mitochondria [224]. Finally, it is not always possible to compare results obtained in different animal models such as, for example, mice and rats, or human and rodents. In fact, it has been seen that effects of TH on some parameters are differently influenced and specie-specific, such as (for example) the activation of the UCP3 gene [225].

In conclusion, much progress has been made in recent years on the actions that iodothyronines exert on the respiratory chain, but much remains to be discovered. For example, what precisely allows mitochondria from animals in different thyroid states to show different morphological characteristics (signature, cristae density, etc.), which may be the basis of some functional characteristics of organelles, is crucial.

### A Final Speculation

What is the goal that lies in the short- and long-term actions? In our opinion, a twofold objective could be achieved: (a) the activation of short-term mechanism would be useful for a rapid response to sudden physiological variations (such as, for example (in animals) a systemic and cellular metabolic adaptation following a sudden and momentary drop in the environmental temperatures); (b) the activation of the long-term one could be functional to a long-lasting cellular metabolic adaptation (for example during the winter or chronic metabolic/physiologic changes of the organism). In the last case the changes may be useful to: (i) produce organelles with higher density in respiratory components; (ii) produce organelles more or less efficient in the ATP synthesis (e.g., with higher or lower UCPs concentration); (iii) regulate the mitochondrial turnover. T2 could be more functional in the first case while T3 in the second.

Elucidation of the mitochondrial molecular mechanisms and downstream targets of THs would provide not only new basic knowledge in the field of thyroid endocrinology and metabolism but also new perspectives to develop therapeutic strategies for a number of high incidence public health issues, such as dysthyroidisms (hypo- and hyperthyroidism), ageing and associated comorbidities (e.g., type 2 diabetes, obesity). Indeed, altered mitochondrial bioenergetics due to pathological and even subclinical changes in circulating TH levels or tissue-specific local action have been associated to the pathogenesis of degenerative diseases such as sarcopenia, liver steatosis, cardiomyopathy, and neurodegeneration, in all of which a strict interconnection has been described between cellular ATP levels, oxidative stress, mitochondrial dynamics, autophagy and substrate metabolism.

## Figures and Tables

**Table 1:** Main general quali-quantitative effects of TH on the mitochondrial respiratory chain.

Parameters	Reported Changes	TH	References
Activity of electron transport chain components	increase	T3, T4	[29,30]
P/O ratio	no change or decrease	T4	[43,44]
Redox slipping	increase	T3	[26] (and references within)
Proton leak	increase	T3, T2	[26,41] (and references within)
Activity of uncoupling proteins (ANT, UCPs)	increase	T3	[31,32,33,52,53,54,55,56,57]
PTP opening	increase	T3	[39,73,74,75,76,77]
Activity of K-glycerophosphate dehydrogenase, succinic dehydrogenase, NADH dehydrogenase and calcium uptake	increase	T2, T3, T4	[34,88]
Cardiolipin-synthase activity	increase	T3	[26] (and references within)
Reduced cytochromes	increase	T3	[26,38] (and references within)
Transcription of nuclear and mitochondrial respiratory genes	increase	T3, T2	[35,36,37,89,90]
Supercomplex aggregation and activity	increase	T3, T2	[90]

## Data Availability

Not applicable.
